# Brain Topology Disruption in Early‐Onset Dementia: Review of Current Findings and the Need for Network Resilience Focused Models

**DOI:** 10.1002/brb3.70903

**Published:** 2025-11-18

**Authors:** Hema Nawani, Sredha S. Sunil, Ranjith Jaganathan, Veeky Baths

**Affiliations:** ^1^ Cognitive Neuroscience Lab BITS Pilani K K Birla Goa Campus Goa India; ^2^ Consciousness Studies Programme National Institute of Advanced Studies Bengaluru India; ^3^ Department of Computer Science and Information Systems BITS Pilani K. K. Birla Goa Campus Zuari Nagar Goa India

**Keywords:** behavioral variant frontotemporal dementia (bvFTD), community resilience, early‐onset dementia (EOD), frontotemporal dementia (FTD), graph network, modularity, network resilience

## Abstract

**Introduction:**

Early‐Onset Dementia (EOD), including Frontotemporal Dementia (FTD), behavioral variant FTD (bvFTD), and Early‐Onset Alzheimer’s Disease (EOAD), presents significant diagnostic and therapeutic challenges due to heterogeneous clinical features and rapid progression. EOD involves distinct patterns of brain network disruption, measurable through graph‐theoretical analysis.

**Methods:**

We reviewed 23 studies applying graph theory to electroencephalography (EEG), functional MRI (fMRI), diffusion tensor imaging (DTI), and fluorodeoxyglucose positron emission tomography (FDG‐PET) in EOD populations. Metrics included global and local efficiency, small‐world properties, modularity, hub connectivity, and rich‐club organization.

**Results:**

EOD demonstrates widespread topological disruption, including reduced global and local efficiency, hub vulnerability, and modular fragmentation. Subtype‐specific patterns include compensatory efficiency increases and parietal‐to‐frontal hub shifts in FTD; disruption of hub connectivity and modular integrity within the salience network in bvFTD; and pronounced deterioration of the default‐mode network in EOAD. Small‐world properties are generally preserved in early stages, reflecting initial compensatory reorganization that precedes system‐wide collapse.

**Conclusion:**

Graph‐theoretical analysis reveals characteristic topological disruptions in EOD. However, most studies rely on static measures, limiting insight into dynamic network vulnerability. Incorporating network resilience based computational models, longitudinal designs, and standardized analytical pipelines could clarify network failure mechanisms, uncover latent fragilities, and guide targeted interventions.

## Graphical Abstract

This review discusses findings from 23 neuroimaging studies using Electroencephalography (EEG), resting‐state Functional Magnetic Resonance Imaging (rs‐fMRI), Diffusion Tensor Imaging (DTI), and Positron Emission Tomography (PET) to examine brain network topology in Early‐Onset Dementia (EOD), including Frontotemporal Dementia (FTD), behavioral variant Frontotemporal Dementia (bvFTD), and Early‐Onset Alzheimer's Disease (EOAD). Findings indicate heterogeneous alterations in global efficiency, modularity, and hub organization, with small‐worldness often preserved. Current gaps involve the lack of network resilience modeling and limited understanding of cognitive reserve mechanisms. Future directions emphasize resilience‐based frameworks to capture dynamic network reconfiguration and individual variability in disease progression.

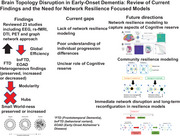

## 1 Introduction

**FIGURE 1:**
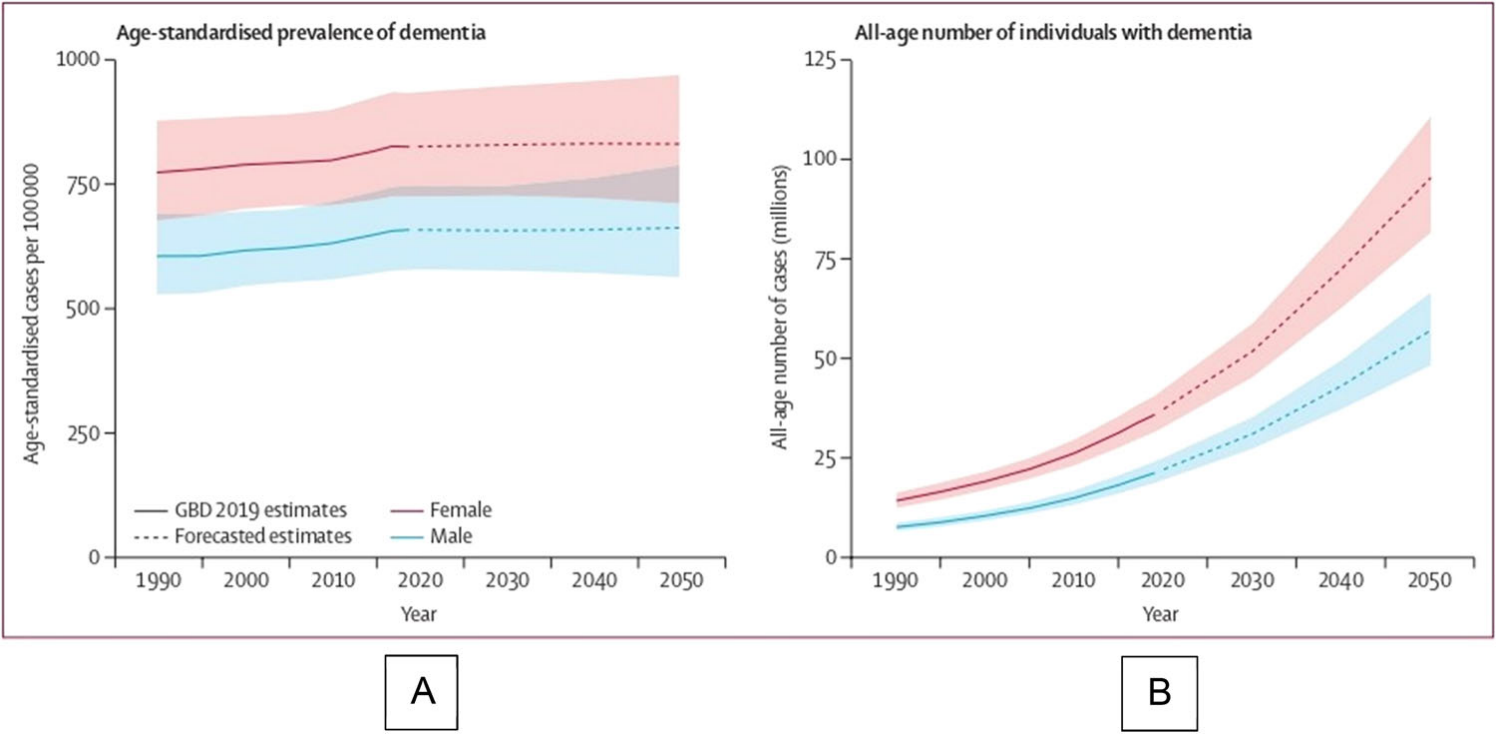
GBD 2019 Dementia Forecasting Collaborators. (2022). Estimated trends in global age‐standardized dementia prevalence (A) and all‐age number of cases (B) with 95% uncertainty intervals, 2019–2050. Reprinted from: Estimation of the global prevalence of dementia in 2019 and forecasted prevalence in 2050: an analysis for the Global Burden of Disease Study 2019, *Lancet Public Health, 7(2)*, e105–e125, under Creative Commons Attribution License (CC‐BY).

Dementia is becoming a major public health issue, with its global prevalence rising due to aging populations (GBD 2019 Dementia Forecasting Collaborators 2022). While late‐onset dementia (LOD) has garnered most attention, early‐onset dementia (EOD), which includes conditions like Frontotemporal Dementia (FTD), behavioral variant (bvFTD), and early‐onset Alzheimer's disease (EOAD), presents unique challenges (Figure 1A,B). EOD, defined by neurodegeneration before age 65 (Rossor et al. 2010), often involves diverse, overlapping cognitive impairments that are poorly understood, leading to frequent misdiagnosis (Hershey 1996). This results in inadequate diagnosis, care, and support, particularly in countries without sufficient services (Rossor et al. 2010, van Vliet et al. 2010). Unlike late‐onset Alzheimer's, EOD presents with a range of non‐amnestic symptoms, including attention deficits, executive dysfunction, language impairments, and visuospatial difficulties (Jacobs et al. 1994, Koedam et al. 2010).

EOAD patients typically show multi‐domain cognitive impairments, including visuospatial, language, attention, and executive function deficits, with notable precuneus atrophy (Karas et al. 2007, Koss et al. 1996). Frontotemporal Lobar Degeneration (FTLD), a group of neurodegenerative conditions affecting the frontal and temporal lobes, includes FTD, which presents with behavior, language, or executive function changes. FTD subtypes, such as bvFTD, svPPA, nfvPPA, and rtvFTD, vary in clinical presentation (Antonioni et al. 2023). bvFTD is primarily characterized by behavioral changes, including personality shifts and executive dysfunction, with memory disturbances often being less pronounced, accompanied by anterior cingulate and frontal insular degeneration (Neary et al. 1998, Seeley 2008). The clinical overlap between EOAD and bvFTD complicates diagnosis, often leading to misdiagnosis, even in specialized centers (Rascovsky et al. 2011). This complexity and rapid progression make early, accurate diagnosis of EOD challenging.

Neuroimaging modalities like electroencephalography (EEG), diffusion tensor imaging (DTI), resting‐state fMRI (rs‐fMRI), and fluorodeoxyglucose positron emission tomography (FDG‐PET) have enabled a more comprehensive understanding of how structural and functional connectivity are disrupted in these neurodegenerative disorders (Filippi et al. 2013, Gour et al. 2014, Hafkemeijer et al. 2015). Recent advances in neuroimaging, particularly the application of graph theory to structural and functional brain networks (Tijms et al. 2013), have provided valuable tools for investigating the brain topology in EOD. Together, these modalities offer powerful insights into the pathophysiology of EOD.

Graph network allows characterization of brain networks through metrics that capture integration (e.g., global efficiency), segregation (e.g., local efficiency and clustering coefficient), modular organization (modularity), and nodal importance (degree centrality, betweenness centrality, hubness) (Table S1). Across the 23 studies reviewed, several consistent trends and challenges emerge. We categorize findings by subtype, identify the most commonly used graph metrics, and give a network statistics summary.

This review is organized as follows: First, we summarize network metrics such as global and local efficiency, modularity, hub disruption, and rich club architecture across 23 studies on EOD (Section 3), highlighting key findings and methodological limitations (Section 4). Next, we propose future directions and introduce the conceptual framework of network and community resilience (Section 5) and discuss the implications of incorporating resilience metric (Section 5).

## 2 Methods

### 2.1 Search Terms and Selection Criteria

**FIGURE 2:**
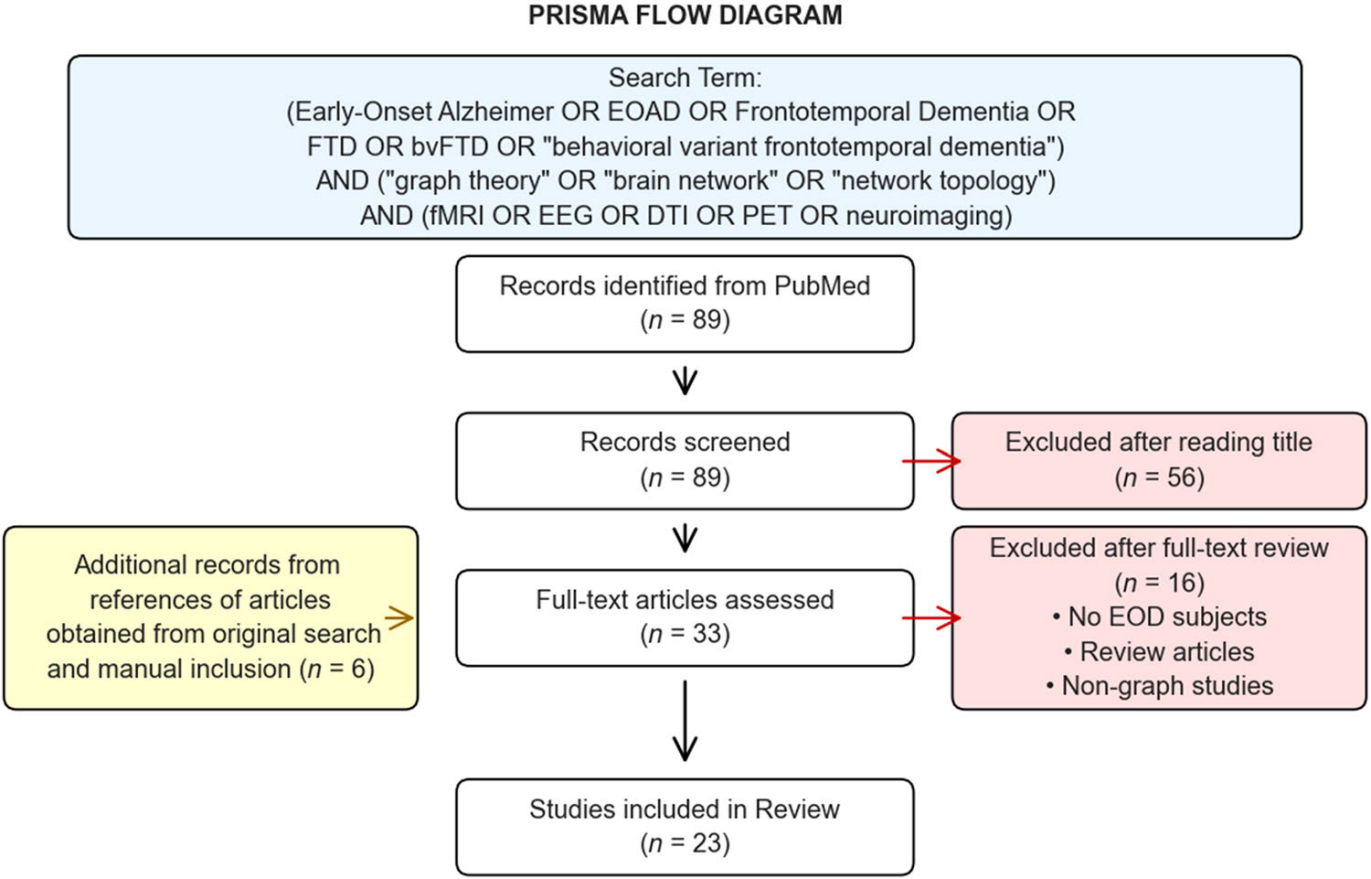
Preferred reporting items for systematic reviews and meta‐analyses (PRISMA) flow diagram showing the selection process of studies included in the review.

A comprehensive search was conducted for relevant articles using the following key search terms: (early‐onset Alzheimer OR EOAD OR frontotemporal dementia OR FTD OR bvFTD OR “behavioral variant frontotemporal dementia”) AND (“graph theory” OR “graph analysis” OR “graph‐based” OR “brain network” OR “network topology”) AND (fMRI OR EEG OR DTI OR PET OR neuroimaging) in PubMed. After careful consideration, 23 articles were included in this review Figure 2. The details of studies included in the review are mentioned in Table 1A,B.

**TABLE 1A:** Studies incorporating EEG, rsfMRI, DTI, FDG‐PET, and graph network approach in EOD.

DOI	Author	Main question	Method	Graph metrics studied	Main results
10.1186/1471‐2202‐10‐101	de Haan et al. (2009)	What are the topological changes in brain networks in AD and FTLD?	EEG	Clustering coefficient, characteristic path length, degree correlation	**AD < HC**: clustering coefficient in lower alpha/beta, path length in lower alpha/gamma, degree correlation in lower alpha **FTLD > HC**: degree correlation in lower alpha, no other significant differences
10.1016/j.neurobiolaging.2016.03.018	Yu et al. (2016)	What are the functional connectivity and network topology alterations in bvFTD, AD, and SCD?	EEG	MST measures, whole brain and regional PLIs	**AD < bvFTD**: whole‐brain PLI connectivity in delta/alpha **bvFTD < AD**: whole‐brain PLI connectivity in theta Preserved posterior connectivity in alpha, abnormal frontal connectivity in bvFTD In contrast to AD and SCD groups, the bvFTD patients showed abnormal functional connections between the left and right hemispheres of the brain because of the disruption of the frontal cluster: the frontal nodes were merged into a left posterior cluster, a right central–posterior cluster, and a frontal–occipital cluster.
10.1016/j.neurobiolaging.2021.10.016	Franciotti et al. (2022)	How does modularity in brain networks change in FTD and AD?	EEG	Hubs, modularity	**FTD**: parietal “main hub” of HC and AD patients lost in the FTD patients at the dementia onset, substituted by frontal “provincial hubs” and local “small worlds.” **AD**: No change in global network organization FTD exhibited relatively preserved function in the occipital region, which is primarily responsible for processing visual information
10.1186/s12868‐024‐00877‐w.	Wu et al. (2024)	What are the alterations in brain network connectivity in AD and FTD?	EEG	Mean PLI, clustering coefficient, global efficiency, local efficiency	**AD and FTD < HC**: clustering coefficient, global efficiency, and local efficiency in the alpha band **AD and FTD > HC**: clustering coefficient, global efficiency, and local efficiency in theta band **AD (Occipital Region)**: Increased theta band node degree, decreased alpha band clustering coefficient and local efficiency, not observed in FTD.
https://arxiv.org/abs/2409.16823	Ranjan et al. (2024)	EEG‐based network organization in AD and FTD	EEG	CPTE, clustering coefficients, eigenvector centrality	**FTD > AD**: Higher connectivity in delta, theta, and gamma bands due to lower neurodegeneration. **CPTE**: Network parameters classify AD and FTD with 87.58% accuracy, gamma band shows highest accuracy (92.87%).
10.1212/WNL.0b013e31829a33f8	Agosta et al. (2013)	What are the topological changes in bvFTD using rsfMRI?	rsfMRI	Clustering coefficient, path length, clustering coefficient, mean network degree, global efficiency, assortativity, hubs	**bvFTD < HC**: global efficiency, nodal centrality in the frontal lobe, interregional connectivity between frontal, temporal, occipital, insular, and subcortical regions, **bvFTD > HC**: trend toward higher centrality in inferior parietal regions
10.1017/S1355617715000703	Sedeño et al. (2016)	How is network centrality disrupted in bvFTD and level of involvement of frontotemporoinsular network in social–executive performance?	rsfMRI	Network Centrality (NC)	**bvFTD < HC** bilateral and right side of the frontotemporoinsular network, associated with executive functions and social cognition
10.1002/hbm.23627	Sedeño et al. (2017)	How consistent are functional connectivity biomarkers for bvFTD across centers and different socio‐cultural background?	rsfMRI	Seed analysis, interregional connectivity, characteristic path length, average clustering coefficient, degree, closeness centrality, betweenness centrality	**bvFTD < HC**: node centrality **bvFTD**: Temporal and frontal regions consistently showed less interconnected and integrated nodes across centers, reflecting the characteristic pathophysiology of bvFTD.
10.1212/WNL.0000000000004577	Filippi et al. (2017)	How do functional network architectures in EOAD and bvFTD differ?	rsfMRI	Clustering coefficient, characteristic path length, mean network strength, and local efficiency, regional connectivity analysis	**EOAD < HC**: lower mean nodal strength, local efficiency, clustering coefficient, and longer path length. **bvFTD**: Relatively preserved global functional brain architecture. **bvFTD < HC**: Reduced nodal strength in the frontoinsular lobe and focal altered connectivity in frontoinsular and temporal regions. **EOAD < bvFTD** (mean nodal strength, local efficiency, and mean path length of the parietal lobe)
10.1155/2018/9684129	Reyes et al. (2018)	How does functional connectivity differ in FTD variants—bvFTD, semantic (svPPA), and nonfluent variant (nfvPPA)?	rsfMRI	Network basic statistics and topological measures	**bvFTD**: Hubs in the frontal, limbic, and basal ganglia are compromised. **nfvPPA** < **bvFTD** and **HC**: global efficiency, disconnection in parietal regions.
10.3389/fnins.2019.00211	Saba et al. (2019)	How do global connectivity and topological changes in bvFTD manifest when analyzed using MST?	rsfMRI	MST global and local metrics	**bvFTD < HC**: Fragmented global network, disrupted frontal–temporal connections.
10.1016/j.neurobiolaging.2018.12.009	Rittman et al. (2019)	To investigate the contribution of functional network resilience to preserved cognition in presymptomatic genetic frontotemporal dementia?	rsfMRI	Global efficiency, mean connection strength, closeness centrality	**FTD < Gene negative group < Gene carrier FTD**: Mean connection strength **Gene carrier FTD > Gene negative group**, **FTD**: Global efficiency **Gene carrier FTD > FTD**: connectivity in frontal lobes, temporal lobes, occipital lobes, and cingulate cortices, cerebellum, and insula cortices, efficiency in occipital cortex
10.1186/s13195‐020‐00752‐w	Ng et al. (2021)	How do topological changes in AD and bvFTD networks differ and underpin cognitive and social–emotional functional deficits?	rsfMRI	Degree centrality, nodal efficiency, within‐module degree, and participation coefficient	**AD**: Disrupted default and control network integration. **bvFTD**: Disrupted salience network integration
10.1007/s11682‐021‐00470‐3	Zhou et al. (2021)	What are the neuropathological mechanisms in EOAD and LOAD?	rsfMRI	DC mapping and EC mapping	**EOAD < YC**: DC in the middle temporal gyrus (MTG), parahippocampal gyrus (PHG), superior temporal gyrus (STG); EC in MTG, PHG, and postcentral gyrus. **LOAD < OC**: DC in the STG and anterior cingulum gyrus **LOAD > OC**: DC in the middle frontal gyrus. **LOAD**: No significant difference in EC compared to OC.
10.1007/978‐3‐319‐11182‐7_2	Daianu et al. (2014)	How is the 'rich club' network disrupted in bvFTD and EOAD?	DTI	Rich club	**bvFTD**: Disrupts both nodal and global network organization in low‐ and high‐degree brain regions. **EOAD**: Targets global connectivity, primarily affecting fiber density in high‐degree regions forming the rich club network.
10.1002/hbm.23069	Daianu et al. (2015)	How does rich club and anatomical network disruption differ in probable bvFTD and EOAD?	DTI	Rich club	**EOAD**: overall less disruption, with more involvement of richly interconnected posterior brain areas associated with cognitive alterations **bvFTD**: Greater disruption, particularly in the rich club and more peripheral alterations in medial frontal areas, correlating with behavioral and socioemotional deficits.
10.1117/12.2082352	Daianu et al. (2015)	How are brain hubs and communication altered in bvFTD and EOAD?	DTI	hubs	**High‐cost backbone**: Composed of fiber density and minimally short pathways, accounting for 81%–92% of overall brain communication in all diagnostic groups. **bvFTD**: Altered global and regional organization of high‐cost and high‐capacity brain networks compared to healthy controls. **EOAD**: Relatively preserved organization of high‐cost and high‐capacity networks compared to controls.
10.1016/j.neurobiolaging.2023.01.004	Chu et al. (2023)	To investigate the clinical relevance of WM topological alterations in probable bvFTD	DTI	(1) normalized characteristic path length, normalized clustering coefficient, and small‐worldness, global efficiency, and local efficiency (2) nodal clustering coefficient, nodal shortest path length, nodal efficiency, nodal local efficiency, and degree centrality.	**bvFTD**: Disrupted global and local topological organization. **Hub Regions**: Different distribution patterns of hub regions compared to controls. **Nodal Efficiency**: In the right superior orbital frontal gyrus, associated with apathy and disinhibition.
10.3988/jcn.2023.0092	Heo et al. (2024)	How does structural network connectivity and its association with cognitive profile differ in EOAD and LOAD?	DTI, PET	Network topology measures	**EOAD**: More pronounced and widespread impairment of structural network efficiency compared to age‐matched controls. **LOAD**: No significant impairment in network efficiency compared to age‐matched controls.
10.3389/fnagi.2016.00159	Chung et al. (2016)	How does metabolic graph connectivity change in EAD and LAD?	FDG PET	Metabolic connectivity analysis, clustering coefficient, and global efficiency	**EAD**: More extensive and progressive deterioration in metabolic connectivity centered in the cingulate gyri and occipital regions **LAD**: Less pronounced changes in metabolic connectivity compared to EAD, decreased connectivity in the occipital and temporal regions, as well as increased connectivity in the supplementary motor
10.1016/j.cortex.2019.07.018	Malpetti et al. (2019)	How is dysfunctional connectivity manifested in different bvFTD variants (frontal and temporo‐limbic bvFTD variants)	FDG PET	SICE and IRCA analysis, hubs, modularity measures	**bvFTD**: Dysfunctional connectivity patterns observed in frontal, limbic regions, and all major resting‐state networks compared to healthy controls. **Frontal bvFTD Variant**: Characterized by metabolic connectivity alterations in orbitofrontal regions and anterior resting‐state networks. **Temporo‐limbic bvFTD Variant**: Characterized by connectivity alterations in the limbic and salience networks.
10.1186/s13195‐022‐01061‐0	Lee et al. (2022)	(a) Proposed the existence of distinct tau‐propagation networks between Early‐Onset Alzheimer's Disease (EOAD) and Late‐Onset Alzheimer's Disease (LOAD) spectra. (b) Aimed to identify tau‐spreading pathways through data‐driven analysis using longitudinal tau PET imaging data.	tau PET	(1) Directional graph theory regression (2) Multilayer community detection	**Young CU/Early‐Onset CI Group**: Tau spread across three independent, partially overlapping communities, with key regions including the basal temporal regions, left medial and lateral temporal regions, and left parietal regions. **Old CU/Late‐Onset CI Group**: Tau spread continued within major communities, with hub regions emerging in the sequence of bilateral entorhinal cortices, parahippocampal and fusiform gyri, and lateral temporal regions.
10.1002/hbm.26140	Rus et al. (2023)	To identify and validate bFDRP in a new independent population, explore its internal structure and the contribution of structural atrophy to the metabolic pattern.	FDG PET	(1) Multivariate SSM‐PCA procedure for metabolic bFDRP‐SLOV identification Validation and clinical correlations (2) Longitudinal analysis Internal network organization (3) Exploring the relationship between atrophy and metabolic bFDRP‐SLOV pattern (4) Relationship between bFDRP‐SLOV and DMN (5) degree centrality, random graph normalized clustering coefficient, random graph normalized characteristic path length, small‐worldness, and assortativity	**bFDRP in bvFTD**: Identified in 20 bvFTD patients and validated in three cohorts. **Metabolic Pattern**: Hypometabolism in the frontal cortex, insula, cingulate, caudate, thalamus, and temporal poles. **Group Comparison**: Higher expression in bvFTD versus HC correlated with cognitive decline **Network Disruption**: Network disruptions correlated with atrophy, but made a minimal contribution to the metabolic pattern. **DMN Overlap**: Overlap with FDG‐PET‐derived DMN. **Validation**: bFDRP as a diagnostic/prognostic biomarker, independent of structural atrophy and resting‐state network loss. **bvFTD**: Extensive decrease in eigenvector centrality was in the frontotemporal module in contrast to the parieto‐occipital module **bvFTD < HC**: degree centrality **bvFTD > HC**: random graph normalized clustering coefficient, characteristic path length, small‐worldness, and assortativity

Abbreviations: AEC‐c, amplitude envelope correlation (corrected); AD, Alzheimer's disease; bFDRP, bvFTD‐specific multivariate metabolic brain pattern; bvFTD, behavioral variant frontotemporal dementia; CI, cognitively impaired; CPTE, Cross‐plot transition entropy; CU, cognitively unimpaired; DC, degree centrality; DMN, default mode network; DTI, diffusion tensor imaging; EC, eigenvector centrality; EOAD, early‐onset Alzheimer's disease; FDG‐PET, fluorodeoxyglucose positron emission tomography; FTLD, frontotemporal lobar degeneration; HC, healthy controls; IRCA, seed‐based interregional correlation analyses; LOAD, late‐onset Alzheimer's disease; MST, minimum spanning tree; nfvPPA, nonfluent variant primary progressive aphasia; PLIs, phase‐lag index; PPA, primary progressive aphasia; rsfMRI, resting‐state functional magnetic resonance imaging; SCD, subjective cognitive decline; SICE, sparse inverse covariance estimation; SSM‐PCA, sparse subspace mapping‐principal component analysis; svPPA, semantic variant primary progressive aphasia.

**TABLE 1B:** Graph characteristics of studies mentioned in Table 1A.

DOI	Author	Method	Group	FC	Graph Type	Nodes	Network structure	DMN or Salience findings	Global efficiency, Local efficiency, CC, Characteristic Path length	Hubs, centrality	Modularity	Correlation with MMSE
10.1186/1471‐2202‐10‐101	de Haan et al. (2009)	EEG	FTLD (15) AD (20) HC (23)	synchronization likelihood (SL)	B	19	AD—random FTD—ordered		AD—CC (lower alpha and beta) and P (lower alpha and gamma) compared to HC FTLD—no difference from HC in CC, P			In the lower alpha band in AD, normalized characteristic path length (λ) was positively correlated with MMSE score (*r* = 0.50, *p* < 0.05)
10.1016/j.neurobiolaging.2016.03.018	Yu et al. (2016)	EEG	AD (69) bvFTD (48) SCD (64)	PLI, AEC	W	21	AD—line like bvFTD—star‐like SCD—star like		bvFTD—GE preserved AD—GE loss	AD—Increased BC in frontal regions, increased eccentricity in the occipital region compared to SCD bvFTD—no differences in BC compared to SCD bvFTD and SCD—hubs present in O1 and O2 AD—hubs in parietal (P4 and P3) and central (Cz) regions.		
10.1016/j.neurobiolaging.2021.10.016.	Franciotti et al. (2022)	EEG	HC (20) FTD (18) AD (18)	Mutual Information (MI)	B	19	FTD—higher levels of small‐worldness, assortative network AD—did not show increased small‐worldness, disassortative network		FTD—GE, LE, CC increased compared to HC and AD AD—GE decreased compared to FTD	FTD—loss of the parietal main connector hub and appearance of provincial hubs in frontal areas (Fp2 and F4 electrodes) HC and AD—showed a main connector hub in parietal areas (P3 electrode)	FTD—derangement in cortical network modularity possibly, due to dysfunctions in frontal *functional connectivity*	
10.1186/s12868‐024‐00877‐w.	Wu et al. 2024	EEG	AD (36) FTD (23) HC (29)	PLI, AEC	B	19			FTD, AD—GE, LE, and CC increased compared to HC in the theta band FTD—GE, LE, CC decreased compared to HC in the alpha band No differences in P			
https://arxiv.org/abs/2409.16823	Ranjan et al. (2024)	EEG	AD (36) FTD (23) HC (29)	Crossplot Transition Entropy	W	19			AD—lower median CC, compared to FTD patients in the delta and theta bands	AD—lower median SC, and EC values compared to FTD patients in the delta and theta bands		
10.1212/WNL.0b013e31829a33f8	Agosta et al. (2013)	rsfMRI	bvFTD (18) HC (50)	Pearsons	B	90	bvFTD—higher network assortativity compared to HC bvFTD—SW similar to HC	Salience	bvFTD—GE, CC decreased, and P increased compared to HC	bvFTD retention of major “hub” regions in the medial parietal, temporal, and occipital lobes, cortical hubs not noted in the frontal lobes bvFTD—lower nodal centrality predominantly in the frontal lobe, and a trend toward higher nodal centrality predominantly in the inferior parietal cortical regions was seen in bvFTD		
10.1017/S1355617715000703	Sedeño et al. (2016)	rsfMRI	bvFTD (14) fronto insular Stroke (10) HC (12)	Wavelet	B	116		bvFTD: frontotemporol insula pointing to the salience network		bvFTD—reduced NC of the frontotemporoinsular network compared to HC		lack of association between the frontotemporoinsular NC and the MMSE
10.1002/hbm.23627	Sedeño et al. (2017)	rsfMRI	Argentina bvFTD (16) FIS (13) HC (16)	Pearsons	B, W	90			bvFTD—increased P, decreased CC compared to HC	AD—showed alteration in posterior regions without almost any involvement of frontal hubs bvFTD—decreased degree centrality and increased BC compared to HC in frontal‐limbic regions		
			Colombia bvFTD (17) PPA (8) HC (29)									
			Australia bvFTD (12) AD (13) HC (15)									
10.1212/WNL.0000000000004577	Filippi et al. (2017)	rsfMRI	EOAD (37) bvFTD (38) HC (32)	Pearsons		220, Grouped into six anatomic macro‐areas			EOAD decreased LE, CC and increased P compared to HC bvFTD—no global network abnormalities compared to HC			
10.1155/2018/9684129	Reyes et al. (2018)	rsfMRI	bvFTD (50) svPPA (14) nfvPPA (12) HC (32)	Pearsons		AAL			bvFTD—GE significantly increased compared to nfvPPA, nfvPPA—less GE compared to HC not significant	bvFTD—hubs in the limbic system and basal ganglia were compromised in the behavioral variant, apart from frontal networks		
10.3389/fnins.2019.00211	Saba et al. (2019)	rsfMRI	bvFTD (41) HC (39)	Wavelet	MST	116, AAL	bvFTD—linear configuration HC—tree	bvFTD: dysfunctions in DMN, SN, and EN networks		bvFTD: high distance between nodes, low centrality parameter values, and a low exchange information capacity, where the superhighway system in HCs, linking hubs to frontal and temporal brain areas, is replaced by a local (i.e., isolated) network surrounding conserved hubs. new FTD‐specific hub (area #23: Frontal_Sup_Medial_L), absent in HC		
10.1016/j.neurobiolaging.2018.12.009	Rittman et al. (2019)	rsfMRI	FTD (29) Gene carriers (70) Gene negative (86)	Wavelet		500			Gene carrier FTD > Gene negative group, FTD: Global efficiency	FTD: weaker hubs		strong relationships in the FTD group of connection strength with MMSE
10.1186/s13195‐020‐00752‐w	Ng et al. (2021)	rsfMRI	AD (50) bvFTD (14) age and gender matchedHC (47)	Pearsons	W	144		AD had lower integration in the default and control networks bvFTD exhibited disrupted integration in the salience network.		AD—lower degree centrality in the right temporal gyrus, but higher degree centrality in the thalamus and left inferior parietal lobule compared to HC bvFTD—lower degree centrality in the thalamus and insula of SVAN, but higher degree centrality in the dorsal prefrontal cortex of DN and the intraparietal sulcus of CN, relative to HC	bvFTD—lowest degree of integration in the thalamus, more fragmented modules in the salience network, and subcortical regions	
10.1007/s11682‐021‐00470‐3	Zhou et al. (2021)	rsfMRI	EOAD (20) YC (19) LOAD (20) OC (17)	Pearsons				EOAD patients showed both impaired local and global centrality properties, involving the DMN and sensorimotor areas LOAD patients showed an impaired local centrality property involving the DMN		EOAD patients showed both impaired local and global centrality properties, involving the DMN and sensorimotor areas LOAD patients showed an impaired local centrality property involving the DMN **EOAD < YC**: DC in the right middle temporal gyrus (MTG), right parahippocampal gyrus (PHG), left superior temporal gyrus (STG); EC in bilateral MTG, right PHG, and right postcentral gyrus. **LOAD < OC**: DC in the left STG and right anterior cingulum gyrus **LOAD > OC**: DC in the right middle frontal gyrus. **LOAD**: No significant difference in EC compared to OC		Across EOAD patients and YC, the DC value of right MTG, STG was positively associated with MMSE scores, the EC value of the left MTG, PHG was positively associated with MMSE
10.1007/978‐3‐319‐11182‐7_2	Daianu et al. (2014)	DTI	bvFTD (15) EOAD (19) HC (30)		W	68, Desikan‐Killiany atlas				bvFTD—impaired fiber density across both the low‐ and high‐degree‐value regime; a more sparse rich club network EOAD—affects the fiber density of major hubs in the network, the organizational integrity of the high‐degree nodes in the rich club network is, however, relatively preserved. bvFTD—The unnormalized rich club coefficient was lower compared to HC EOAD—unnormalized rich club coefficient similar to HC		
10.1002/hbm.23069	Daianu et al. (2015)	DTI	ProbablebvFTD (20) EOAD (23) Age‐matched HC (37)	streamline	W	68; 34 ROI each hemisphere		bvFTD—Salience network atrophy AD—atrophied posterior DMN		bvFTD—rich club disruption in the medial frontal region EOAD—rich club disruption in the posterior region		MMSE scores decreased with the number of edges for the rich club connections
10.1117/12.2082352	Daianu et al. (2015)	DTI	bvFTD (15) EOAD (19) HC (30)	streamline		68		bvFTD—salience AD—DMN		bvFTD—large loss nodal degree in the superior frontal region compared to HC EOAD—no differences were found for nodal degree compared to HC		
10.1016/j.neurobiolaging.2023.01.004	Chu et al. (2023)	DTI	Probable bvFTD (30) HC (30)	streamline	B	90, AAL	probable bvFTD—preserved small‐worldness	probable bvFTD—salience	probable bvFTD—reduced GE LE, CC, and increased P compared to HC	probable bvFTD‐ lost hubs in left anterior cingulate gyrus, left insula, left hippocampus, left middle temporal gyrus, right inferior orbital frontal gyrus, right precentral gyrus, and right putamen bvFTD—reconfigured hub—bilateral cuneus bvFTD—preserved hubs left putamen, bilateral precuneus, left middle occipital gyrus, bilateral lingual, and right median cingulate and paracingulate gyri		
10.3988/jcn.2023.0092	Heo et al. (2024)	DTI	EOAD (47) LOAD (33) HC (57)	streamlines		90, AAL			EOAD—decreased GE and LE compared to age‐matched HC LOAD—no significant difference compared to age‐matched HC			EOAD—MMSE scores negatively correlated with global path length and positively correlated with global efficiency, averaged local efficiency, and averaged clustering coefficient LOAD—no significant correlations
10.3389/fnagi.2016.00159	Chung et al. (2016)	FDG PET	EOAD (74) LOAD (46) YC (20) OC (13)	metabolic connectivity, Pearson's		90, AAL	EAD—no difference in SW compared to HC LAD reduced small‐worldness compared to OC	EAD ‐metabolic connectivity between the dorsal cingulate gyrus (core region of the DMN), inferior parietal, and occipital regions decreased	EOAD—decreased GE, CC compared to YC LOAD—GE and CC similar to OC			Global efficiency and clustering coefficients were also decreased along with disease severity
10.1016/j.cortex.2019.07.018	Malpetti et al. (2019)	FDG PET	Whole bvFTD (82) Frontal variant bvFTD (35) Temporo‐limbic variant bvFTD (35) HC (82)	metabolic connectivity	SICE	121—14 structural–functional macroareas. AAL		bvFTD—alterations in metabolic connectivity in ADMN, PDMN, ECN, ASN, LIN **Frontal bvFTD**: metabolic connectivity alterations in ADMN, ECN and PDMN Temporo‐limbic bvFTD—ASN, LIN		bvFTD—loss of hubs in medial and lateral frontal and temporo‐occipital cortex, thalamus and cerebellum; reconfigured hubs encompassed orbitofrontal and parietal regions **Frontal variant—**reconfigured hubs ‐orbitofrontal, lateral frontal and cerebellar regions **Temporo‐limbic variant**—reconfigured hubs sparser, localized in orbitofrontal, lateral frontal, parietal and inferior temporal regions.	bvFTD: decreased network modularity compared to HC, broad reallocation of nodes across modules, leading to significant reconfigurations in the modular structure of the fronto‐parietal, limbic‐basal ganglia, and cingulum‐temporal modules	
10.1186/s13195‐022‐01061‐0	Lee et al. (2022)	tau PET	YCU (30) OCU (44) EOCI (15) LOCI (53)		W	Desikan–Killiany atlas		preserved posterior DMN may induce faster propagation of tau pathology in related regions and may partly explain the high tau burden and tau‐providing power of hub regions in the EOCI group.		YCU/EOCI group—basal temporal regions, left medial and lateral temporal regions, and left parietal regions OCU/LOCI group‐ bilateral entorhinal cortices, parahippocampal and fusiform gyri, and lateral temporal regions.	**YCU/EOCI group**, the hubs were found in more diffuse regions, including temporal, limbic, and parietal lobes, three communities seemed to be sustained at the similar periods **OCU/LOCI group**, the hubs were more concentrated in the medial and temporal cortices, three major communities showed distinct rising and fading patterns	
10.1002/hbm.26140	Rus et al. (2023)	FDG PET	Slovenia A bvFTD (20) HC (20)			95—AAL	bvFTD—increased small‐worldness, increased assortativity compared to HC		bvFTD‐CC and P significantly increased compared to HC	bvFTD—decreased degree centrality compared to HC	bvFTD—bFDRP‐SLOV network split into two unconnected modules: a fronto‐temporal module and a parieto‐occipital module.	(1) bFDRP‐SLOV correlation with cognitive decline. (2) MMSE able to explain only 18.7% of total bFDRP‐SLOV variance
			Slovenia B bvFTD (20) HC (20)									
			Slovenia C AD (26) sCJD (16) svPPA(8) nfvPPA (6)									
			Slovenia D bvFTD (6)									
			USA bvFTD (25) HC (22)									
			Germany bvFTD (44) svPPA (16) nfvPPA (12) HC (15)									

Abbreviations: AAL, automated anatomical labeling; AD, Alzheimer's disease; ADMN, anterior default mode network; AEC, amplitude envelope correlation; ASN, anterior salience network; B, binary (graph type); BC, betweeness centrality; bvFTD, behavioral variant frontotemporal dementia; CC, clustering coefficient; CjD, Creutzfeldt–Jakob disease; DC, degree centrality; DMN, default mode network; EC, eigenvector centrality; ECN, executive control network; EEG, electroencephalogram; EOAD, early‐onset Alzheimer's disease; EOCI, Early‐onset cognitive impairment; FC, functional connectivity; FDG PET, fluorodeoxyglucose positron emission tomography; FIS, fronto‐insular Stroke; FTLD, frontotemporal lobar degeneration; GE, global efficiency; HC, healthy controls; LE, local efficiency; LIN, limbic network; LOCI, late‐onset cognitive impairment; MMSE, Mini‐Mental State Examination; MST, minimum spanning tree; OC, old control; OCU, old cognitively unimpaired; P, characteristic path length; PDMN, posterior default mode network; PLI, phase‐lag index; PPA, primary progressive aphasia; PPA, primary progressive aphasia; rsfMRI, resting‐state functional magnetic resonance imaging; SCD, subjective cognitive decline; SICE, spherical independent component estimation; SL, synchronization likelihood; SN, salience network; SW, small‐worldness; W, weighted (graph type); YC, young controls; YCU, young cognitively unimpaired.

## 3 Discussion: Network Breakdown in EOD

This review synthesizes findings from 23 studies using EEG, fMRI, DTI, and FDG‐PET with graph theory to examine network topology in EOD subtypes—FTD, bvFTD, and EOAD. Modalities included five EEG, nine rsfMRI, four DTI, four PET, and one combining DTI and PET (Table 1A, 1B). While connectivity definitions vary by modality, consistent findings emerge: disrupted global and local network properties, reduced hub nodes, and lower modularity, supporting the clinical value of graph analysis in EOD.

Traditional metrics like global efficiency and small‐worldness often miss key dynamics such as compensatory changes. In contrast, local efficiency, hub disruption, and modularity provide deeper insights into network breakdown. Furthermore, a critical limitation of the current literature is that none of the 23 studies we reviewed implemented network resilience analysis or community‐based attack models. While many studies noted hub disruption or modular changes, they did not quantify how networks collapse under progressive stress or attack. We identify this methodological and conceptual gap and introduce resilience metrics (e.g., targeted attack models, LCC decay, and modular breakdown) from adjacent domains (e.g., aging, stroke) that could enhance future EOD research, not as part of the current empirical findings.

Of the 23 studies, 14 used Scale 1 (integration/segregation), 18 examined hubs or centrality (Scale 2), and only 5 assessed modularity (Scale 3) (Table 1A, 1B). Scale 2 dominated. To facilitate comparison, we focused on a core set of commonly used network metrics across studies. While some studies reported additional or study‐specific metrics, these were not included in the quantitative summaries shown in Figure 3A‐C. Most models addressed added or rewired links, yet neglected the connection loss central to neurodegeneration (Tijms et al. 2013) Figure 3a Figure 3b Figure 3c.

**FIGURE 3:**
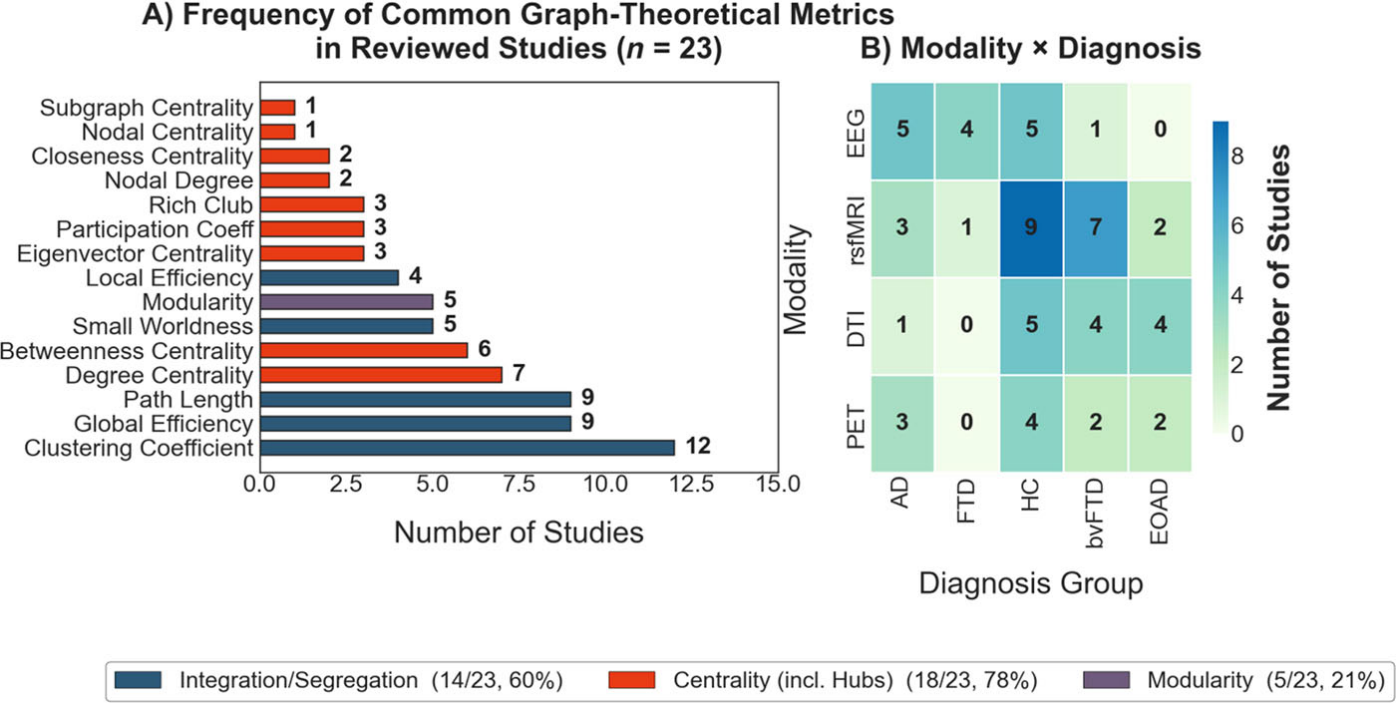
(A) The frequency of selected graph‐theoretical metrics is reported across the 23 studies included in this review. This plot highlights a subset of commonly used metrics—such as global and local efficiency, modularity, and centrality‐based hub measures (e.g., degree, betweenness). Additional metrics reported infrequently or unique to individual studies were not included in this summary. (B) Heatmap of Diagnosis Group and Imaging modality. *One study included subjective cognitive decline (SCD) instead of HC, and one study included gene‐negative: unaffected relatives without mutation for FTD.

**FIGURE 3C:**
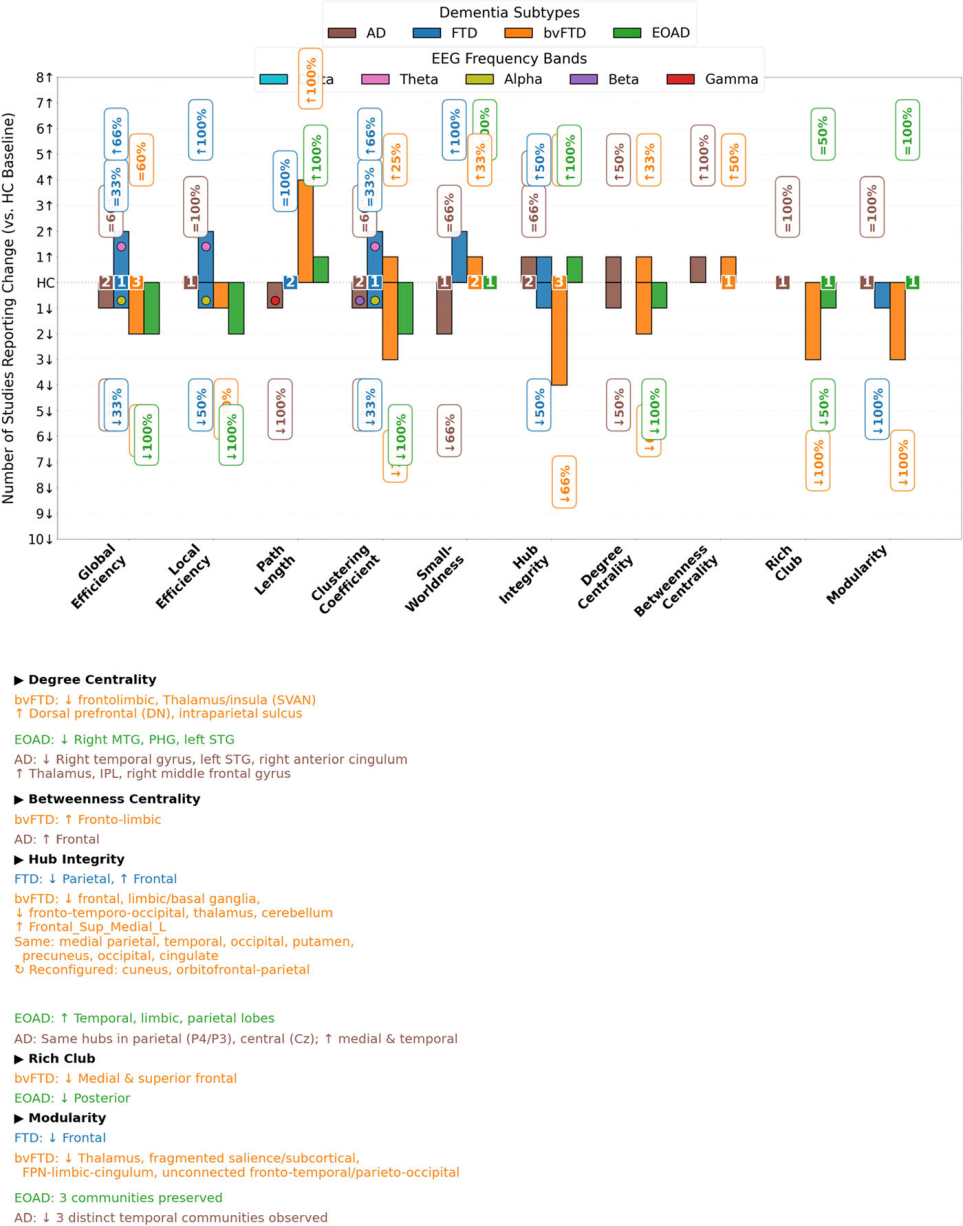
Direction of Reported Metric Changes Relative to HC. For each metric, studies reporting an increase relative to healthy controls were assigned a value of +1, and those reporting a decrease were assigned −1. Percentages were calculated by dividing the number of studies showing a directional change (increase or decrease) by the total number of studies evaluating that metric within the same diagnostic group. Numeric values displayed at the baseline represent the number of studies that reported no significant difference from healthy controls. IPL, inferior parietal lobule; MTG, middle temporal gyrus; STG, Superior temporal gyrus; SVAN, ventral attention network.

### 3.1 Global Efficiency: Limitations in Capturing Network Dysfunction and Compensation in EOD

Global efficiency offers valuable insights into the global organization of brain networks, but its utility in FTD remains uncertain. In the early stages of FTD, global efficiency increases, especially in the theta frequency band, which may reflect compensatory reorganization or cognitive reserve, potentially helping to maintain cognitive function (Rittman et al. 2019, Franciotti et al. 2022, Wu et al. 2024). Global efficiency is calculated based on the shortest‐path assumptions (Bullmore and Sporns 2009), which might not account for the brain's ability to reroute information along less efficient, non‐optimal paths as a compensatory response. This rerouting could temporarily inflate global efficiency values, making it appear that network function is better preserved than it truly is! As such, the increase in global efficiency could mask the underlying dysfunction of the brain, complicating its use as a marker of resilience versus a temporary response to degeneration. As the disease progresses, global efficiency tends to decline in higher‐frequency bands, such as alpha. This decline in global efficiency reflects the breakdown of network efficiency, a hallmark of neurodegeneration. However, global efficiency's inability to account for these dynamic shifts in brain network function may delay diagnosis and obscure the true extent of dysfunction. Therefore, while global efficiency can provide useful insights into the overall network organization, its reliance on simplistic assumptions and failure to capture compensatory dynamics limit its effectiveness as a diagnostic tool in FTD. Global efficiency begins to decline when FTD symptoms appear, signaling a rapid deterioration in brain network organization as the disease progresses from the pre‐symptomatic to symptomatic stages (Rittman et al. 2019). However, no direct correlation was observed between global efficiency and brain atrophy, challenging the notion that changes in brain function (as measured by global efficiency) directly correspond to structural changes early in the disease process. This suggests that while structural changes are important, functional deficits may not always align with them in the early stages of FTD. To understand the network function more closely, global efficiency could be paired with node lesioning approaches in FTD, allowing for a more nuanced understanding of network robustness by identifying critical nodes whose removal significantly disrupts efficiency. Such analysis could reveal latent fragilities that are not captured by static efficiency measures alone, providing a dynamic view of network resilience and collapse thresholds.

In bvFTD, the trajectory of global efficiency is different. Some studies report the preservation of global efficiency (Yu et al. 2016, Reyes et al. 2018), which likely reflects early‐stage compensatory mechanisms. Conversely, several other studies (Agosta et al. 2013) document a decline in global efficiency as the disease severity increases, which aligns with the expected breakdown of network efficiency as the disease progresses. This range of divergent findings, some suggesting compensation and stability, others indicating decline, complicates the understanding of how global efficiency changes across the clinical stages of bvFTD. This variability highlights a methodological paradox, where differences in imaging techniques (e.g., EEG, fMRI, DTI) and patient populations (disease staging and severity) used in these studies complicate the interpretation of global efficiency changes in bvFTD. It will be useful to study network resilience along with global efficiency in bvFTD, to test whether individual differences in network resilience also account for divergent findings.

In EOAD, global efficiency decline is evident early and consistent (Chung et al. 2016), while localized regions like the hippocampus may retain network integrity, creating a local‐global paradox. This preserved connectivity in certain areas temporarily masks broader network dysfunction, complicating the interpretation of brain network changes. Despite some hippocampal integrity (Park et al. 2017), widespread network inefficiencies suggest regional stability doesn't equate to overall health. The rapid global efficiency decline in EOAD, compared to the gradual decline in LOAD, positions global efficiency as a potential early biomarker. Early white matter and metabolic breakdown highlight EOAD's aggressive progression, with localized preservation contrasting with systemic dysfunction (Chung et al. 2016, Heo et al. 2024).

### 3.2 Local Efficiency as a Marker of Compensatory Mechanisms and Regional Decline in EOD

The findings related to local efficiency in FTD, bvFTD, and EOAD offer important insights into the compensatory mechanisms and the regional decline associated with neurodegeneration. In FTD, local efficiency is enhanced in specific regions (e.g., frontal, temporal, parietal, and central regions), particularly in the theta band, suggesting localized compensatory responses (Wu et al. 2024). However, these adaptations are often limited in their scope, as there are reductions in local efficiency in higher‐frequency bands, particularly in the alpha range (frontal and temporal). Interestingly, global efficiency is also increased in FTD, suggesting broader network‐level compensatory mechanisms. This contrasts with AD, where reductions in network efficiency are more pronounced, indicating a different trajectory of network degradation. The preserved function in the occipital region in FTD further highlights the selective vulnerability of brain regions, where sensory regions are more resilient, unlike in AD, where visual‐spatial dysfunction is more common (Geldmacher 2003).

In bvFTD, local efficiency is reduced in the frontal regions critical for executive functions, aligning with the clinical presentation of cognitive and behavioral impairments (Chu et al. 2023). In contrast, preserved local efficiency (Filippi et al. 2017) is also observed. As the disease progresses, frontal areas experience a reduction in local functional connectivity, contributing to symptoms such as apathy, social withdrawal, and impaired decision‐making (Yu et al. 2016, Agosta et al. 2013, Sedeño et al. 2016, Sedeño et al. 2017, Malpetti et al. 2019).

EOAD is characterized by widespread reductions in local efficiency, especially within the DMN and sensorimotor networks, correlating with more severe cognitive and functional deficits (Heo et al. 2024). Unlike bvFTD, where sensory resilience offers some functional preservation, EOAD's systemic local efficiency collapse reflects a more aggressive progression of the disease. Key regions such as the right angular gyrus (ANG), right median cingulate and paracingulate gyrus, right hippocampus, bilateral inferior parietal lobule (IPL), right inferior temporal gyrus (ITG), bilateral middle occipital gyrus (MOG), bilateral middle temporal gyrus (MTG), show substantial reductions in local efficiency among others regions, correlating with the cognitive impairments seen in EOAD, including memory loss, motor difficulties, and executive dysfunction (Heo et al. 2024). Compared to LOAD, EOAD shows a broader and more extensive reduction in local efficiency. This widespread breakdown in local efficiency across the brain's networks suggests that EOAD may lead to faster or more severe network dysfunction, contributing to the more rapid cognitive decline observed in EOAD. Tau pathology, rather than amyloid burden, is identified as the primary factor driving network disruptions in both EOAD and LOAD, underlining the critical role of tau in neurodegeneration. The less pronounced difference between LOAD and older controls (OC) compared to EOAD and younger controls (YC) may partly reflect age‐related reductions in network efficiency. These findings underscore EOAD's aggressive nature and its association with systemic network breakdowns, highlighting the importance of tailored interventions targeting tau pathology to mitigate rapid cognitive decline (Heo et al. 2024).

### 3.3 Small‐World Network Properties in Capturing Disease‐Specific Patterns of Vulnerability and Selective Network Degeneration

Despite their utility in assessing brain network organization, small‐world properties have limitations in capturing the full extent of neurodegenerative changes. Small‐world properties, which reflect an optimal balance between local specialization and global integration, are paradoxically preserved in some EOD subtypes despite declining functional outcomes. FTD reveals an assortative network structure, with highly connected regions predominantly linking to other highly connected areas (Franciotti et al. 2022). Early in FTD, this assortativity supports connectivity within critical networks, such as the salience and frontal networks. In FTD, small‐world properties are enhanced in early stages, suggesting that the brain reorganizes to optimize local communication and compensatory processes (Franciotti et al. 2022). However, this enhancement of small‐world organization does not translate to functional recovery, as individuals continue to experience cognitive decline. Unlike in AD, where small‐world properties are disrupted due to the relative stability of parietal hubs and progressive frontal atrophy, the frontal clustering in FTD underscores the selective vulnerability of this disease to frontal degeneration. This decoupling of structural network metrics from functional outcomes highlights that small‐world measures may fail to capture the complexities of disease‐specific compensatory dynamics, where localized network efficiency comes at the expense of broader cognitive and behavioral deficits. This presents a paradox of structural optimization without functional improvement, where the brain's efficient organization at the local level is insufficient to prevent the collapse of global cognitive functions.

Similarly, in bvFTD and EOAD, small‐world properties are maintained, even as global and local efficiency decline (Agosta et al. 2013, Chung et al. 2016, Chu et al. 2023).

### 3.4 Adaptation and Breakdown of Brain Hubs in EOD: A Paradoxical Shift in Network Function

Hubs are essential nodes that optimize information processing and strengthen a network's robustness against random failures (Pastor‐Satorras and Vespignani 2001). However, they can also act as bottlenecks, as their removal may fragment the network into isolated components. Notably, hubs are integral to epidemic spread, making their analysis crucial for understanding disease transmission within networks (Daianu et al. 2015). Hubs show selective vulnerability in EOD, in FTD, for example, parietal hubs are replaced by frontal hubs (Franciotti et al. 2022). Despite the brain's effort to preserve network connectivity, these newly assigned hubs are less efficient, which contributes to cognitive decline (Franciotti et al. 2022).

In bvFTD, hub vulnerability is characterized by a locally propagating white matter disorder focused on the regions of the frontal cortex, whereas in EOAD, hub vulnerability may be a globally propagating white matter disorder where low‐degree nodes are removed from the overall brain network and may cause the structural and potentially functional damages, most evident in the later stages of the disease (Saba et al. 2019). In bvFTD, frontal hubs, especially in the superior frontal gyrus, are highly susceptible to damage due to white matter degeneration (Saba et al. 2019). These hubs are crucial for global efficiency and the integration of executive and emotional functions. Their impairment results in a significant reduction in global efficiency, leading to the hallmark executive dysfunction seen in bvFTD (Agosta et al. 2013). Recent MST‐based rs‐fMRI studies have demonstrated that bvFTD is associated with global fragmentation of the functional network backbone and severe disruption of information‐flow highways, particularly within the frontotemporal regions (Saba et al. 2019). Compensatory reorganization occurs in regions like the cuneus and parietal lobes, but these sensory and spatial regions cannot fully replicate the integrative functions of frontal hubs, such as executive planning and emotional regulation (Malpetti et al. 2019, Rus et al. 2023), revealing a critical limitation in the brain's compensatory mechanisms. This shift highlights the paradox of sensory compensation, where regions typically less involved in global integration are tasked with maintaining functionality, yet struggle to support higher‐order processes. While certain brain hubs remain intact, overall network connectivity is diminished, resulting in longer node distances, reduced centrality, and decreased global integration. Additionally, large‐scale node reallocation occurs, with significant reconfigurations in the fronto‐parietal, limbic‐basal ganglia, and cingulum‐temporal modules, highlighting disruptions in modular organization (Malpetti et al. 2019). Reyes et al. (2018) reported that hubs within the frontal, limbic, and basal ganglia regions are compromised in bvFTD. Similarly, Sedeño et al. (2016) noted that the reduction in network centrality was specific to the frontotemporoinsular network, consistent with early atrophy patterns, supporting the potential of frontotemporoinsular networks as biomarkers.

In EOAD, tau and amyloid pathology disrupt local hub connectivity, particularly in the medial temporal lobe (Zhou et al. 2021). The default mode network (DMN) and other core networks may redistribute connectivity or leverage alternative pathways to maintain global integration (Zhou et al. 2021, Daianu et al. 2016). However, as local hub disruptions progress, global connectivity eventually deteriorates, leading to widespread cognitive and functional impairments (Gour et al. 2014).

This interplay between local hub disruption and global network compensation underscores the importance of early interventions aimed at stabilizing hub functionality and slowing the spread of pathology. Moreover, it highlights the need for advanced neuroimaging techniques to track the transition from local to global network dysfunction, providing critical insights into disease progression and potential therapeutic windows.

### 3.5 Rich Club Network in bvFTD and EOAD

No study included in this review reported a rich club network in FTD. Disruptions in the rich club network highlight key differences in the pathology of bvFTD and EOAD, affecting global brain communication. In bvFTD, degeneration primarily targets high‐degree nodes within the rich club, particularly in regions critical for executive function and social behavior, leading to a fragmented network (Daianu et al. 2016). This disruption impairs both local and global nodes, hindering long‐range communication and contributing to cognitive and behavioral deficits. In contrast, EOAD shows less severe disruption in the early stages, with high‐degree nodes largely intact despite fiber density loss in major hubs. This suggests that while amyloid and tau accumulation impact local connections, global communication remains relatively preserved early in the disease (Daianu et al. 2015).

Furthermore, bvFTD primarily affects peripheral nodes, with 78% of network nodes—including both rich club and local components severely disrupted (Daianu et al. 2014). The local connections between these nodes are most affected, reflecting impaired network integration. In EOAD, only 28% of the connectome nodes are affected, with disruptions mainly in the rich club nodes and their connections. While both diseases impair the weighted rich club network, further research is needed to determine whether these disruptions are due to the high topological value of rich club nodes or if disease processes originate from or propagate to centrally located nodes (Daianu et al. 2014). Longitudinal studies are crucial to better understand how dysfunction spreads across the network, providing insight into disease progression.

### 3.6 Modular Breakdown and Cognitive Decline in FTD, bvFTD, and EOAD

Disruptions in brain modularity are a key feature of neurodegenerative diseases, playing a significant role in the progression of cognitive decline. Understanding these modular changes could improve diagnostic accuracy and guide the development of targeted interventions. However, only 5 out of 23 studies on EOD have explored the role of modularity. In the case of FTD, there is a notable decline in modularity, particularly in the frontal regions (Franciotti et al. 2022).

In bvFTD, low modularity is linked to disrupted connectivity between key brain modules (Daianu et al. 2016). Increased network segregation is especially evident in the salience network, which impacts the thalamus, contributing to its disintegration (Ng et al. 2021). Healthy controls typically exhibited five distinct brain modules, but bvFTD was characterized by only four modules, with a significant reorganization of nodes across these modules. This reconfiguration notably affects the fronto‐parietal, limbic‐basal ganglia, and cingulum‐temporal modules (Malpetti et al. 2019). Moreover, the brain network in bvFTD becomes split into two isolated modules: a fronto‐temporal module and a parieto‐occipital module. This disconnection is further compounded by a sparse increase in intra‐module connections within the occipito‐vermal region of the parieto‐occipital module, highlighting a reduction in overall network integration (Rus et al. 2023). From a resilience perspective, this may indicate a loss of flexibility and reduced capacity for functional rerouting: a hallmark of reduced network resilience, this can be tested with the community attack resilience approach discussed above.

In EOAD, modularity is disrupted compared to age‐matched controls, with these changes becoming more pronounced as the disease progresses (Chung et al. 2016). Modularity breakdown in EOAD leads to the spread of community hubs across widespread cortical regions, including the temporal, limbic, and parietal lobes. This extensive disruption may accelerate tau propagation, contributing to the high tau burden in this group. In contrast, LOAD shows more localized hubs in the medial and temporal cortices, with tau spreading sequentially from the entorhinal cortex to lateral temporal areas, resulting in less widespread tau spread compared to EOAD (Lee et al. 2022). These findings suggest that modular breakdown differs across neurodegenerative diseases, with EOAD showing a more widespread disruption of modularity, while LOAD and bvFTD exhibit more localized changes.

A formal quantification of this vulnerability through targeted attack simulations and community resilience modeling could provide novel biomarkers for disease progression and targets for theory‐guided perturbations via transcranial direct current stimulation (tDCS) and transcranial magnetic stimulation (TMS) neuromodulatory interventions.

## 4 Limitations and Methodological Gaps in Current Literature

A key limitation of current graph‐theoretical studies in EOD is their reliance on static metrics without modeling dynamic vulnerability. Despite clear evidence of early hub loss and modular breakdown in EOD, none of the 23 studies included in this review implemented resilience modeling, simulated targeted attacks, or quantified collapse thresholds. Another challenge is the lack of standardized methodologies across studies. Variability in parcellation schemes, thresholding techniques, and graph theory metrics hinders comparability between results. These methodological differences, such as variations in threshold settings to avoid false‐positive connections and in how hubs are identified, introduce biases that limit meaningful comparisons (Drakesmith et al. 2015). Standardizing approaches, such as uniform parcellation schemes, is essential to improve reproducibility and facilitate the integration of findings across different imaging modalities (Fornito et al. 2010). Using weighted network measures and minimum spanning trees (MSTs) is a promising strategy to standardize topological comparisons, as MSTs maintain consistency in graph structure by linking nodes with minimal cost (Çiftçi 2011).

## 5 Future Directions

### 5.1 Network and Community Resilience Modeling in EOD: Beyond Static Topology

Conventional graph‐theoretical models have been instrumental in characterizing brain networks by representing regions as nodes and their structural or functional interactions as edges (Table S1). These models quantify properties such as efficiency, path length, clustering, modularity, and hub centrality revealing, disrupted global efficiency and community structure across neurodegenerative conditions (Zhou and Seeley 2014, Bullmore and Sporns 2009, van den Heuvel and Sporns 2013). However, these measures are largely static and descriptive, offering limited insight into how brain networks respond dynamically to progressive damage.

This limitation is particularly relevant in EOD, where neurodegeneration often targets high‐degree hubs and connector nodes early, precipitating rapid, cascading network failure (Filippi et al. 2013, Gour et al. 2014). It is unlikely that brain networks in EOD retain intact resilience mechanisms. To address this, network resilience models introduce dynamic simulations of node loss either randomly or through targeted attacks offering a more functional measure of network integrity under stress. Recent studies on complex systems show that simulating diverse attack strategies can reveal latent network vulnerabilities and help identify critical points of failure (Al Musawi et al. 2023), reinforcing the utility of resilience modeling in brain networks.

A particularly informative resilience metric is the largest connected component (LCC), representing the size of the largest subnetwork that remains interconnected during iterative node removal. In resilient networks, the LCC shrinks gradually; in fragile networks, it fragments abruptly. Argiris et al. (2024) define network resilience as “the iteration of the steepest slope in the LCC decay curve” during targeted attack (Figure 4A). In other words, resilience is not just about whether the network breaks, but *how quickly and severely* it does when critical nodes are removed.

**FIGURE 4A:**
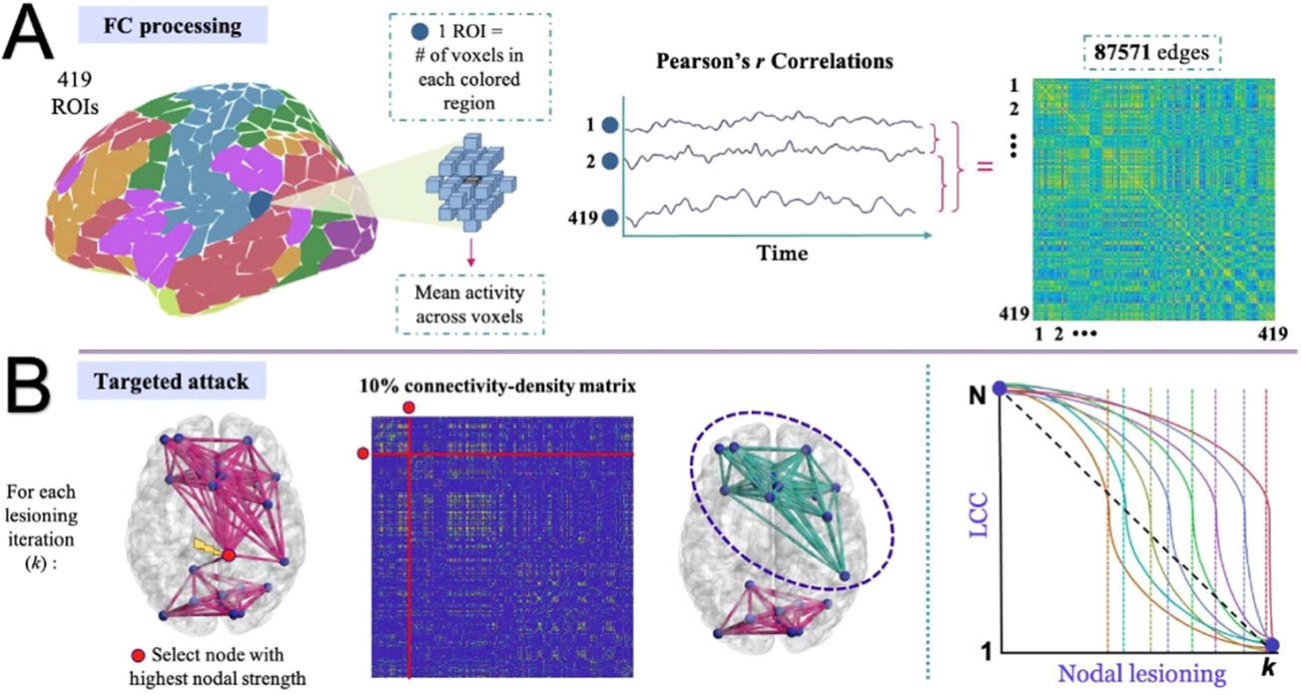
Schematic of functional connectivity (FC) matrix generation and application of targeted attack. Argiris et al. (2024) depict the methodology for quantifying brain resilience using targeted attack simulations on resting‐state functional connectivity networks. Nodes are sequentially removed based on descending nodal strength, and the decay of the largest connected component (LCC) is tracked. The steepest slope of LCC decay is used as a resilience index, with more resilient networks maintaining large‐scale connectivity across more lesioning iterations. The different colored lines in the LCC versus Nodal Lesioning plot represent individual subjects from the study. Reprinted with permission from: Argiris et al. (2024). Brain resilience to targeted attack of resting BOLD networks as a measure of cognitive reserve [Preprint]. Research Square. https://doi.org/10.21203/rs.3.rs‐5356022/v1.

In cognitively healthy older adults, Argiris et al. (2024) reported that delayed or gradual LCC collapse predicted preserved fluid reasoning over 5 years, despite cortical thinning. This positions network resilience as a plausible neural correlate of cognitive reserve: the brain's capacity to adaptively reorganize under strain. While their study focused on aging, the implications for EOD are clear. If resilience supports preserved cognition in healthy aging, its failure in EOD may explain the accelerated and multidomain decline observed, even when atrophy appears moderate. In such cases, early hub loss likely triggers a steep LCC breakdown signaling a tipping point where global communication fails before traditional graph metrics detect the dysfunction.

Beyond global network resilience, community‐level resilience evaluates ability of the brain's modular structure to withstand attack (Jao et al. 2019). Tao and Rapp (2021) showed that connector hubs: nodes linking different modules are critical for maintaining this structure. Their loss leads to greater disruption than random damage, particularly in EOD, where these hubs may be early targets. Community resilience can be understood through two lenses: intercommunity resilience, which assesses the network's ability to maintain integration between modules, and intracommunity resilience, which evaluates a module's ability to function despite node loss (Figure 4B). These dimensions are especially relevant in EOD, where pathology targets networks like the DMN in EOAD or the salience network in bvFTD.

**FIGURE 4B:**
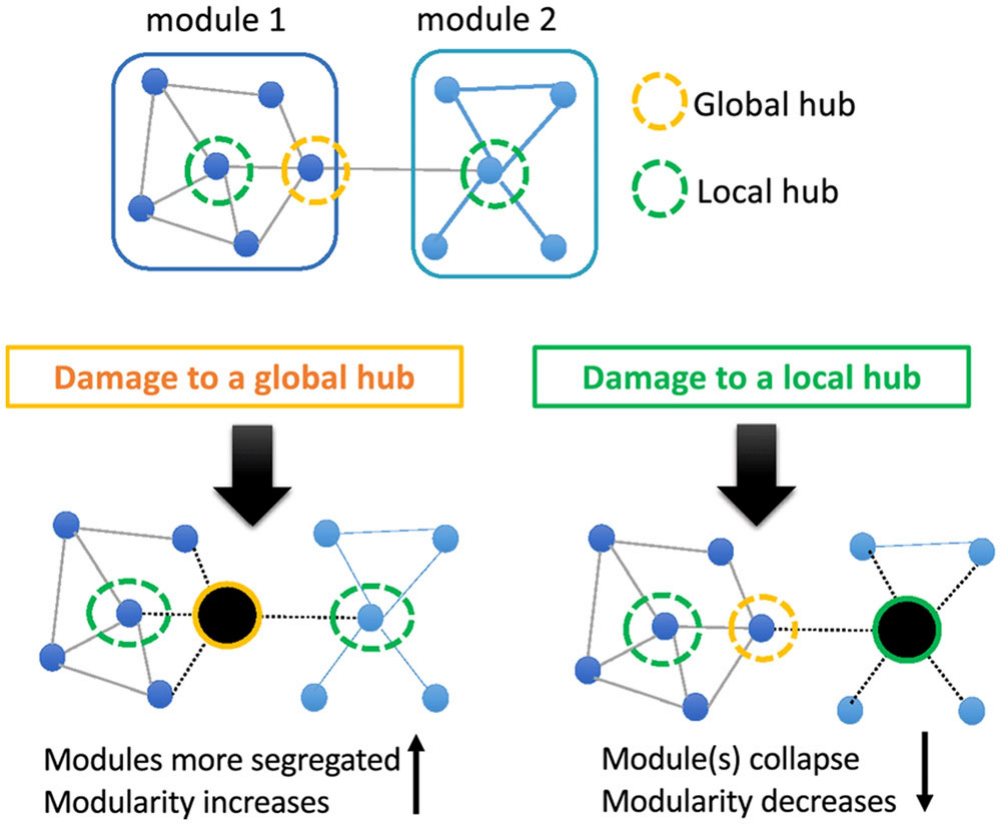
Impact of Focal Lesions on Brain Modularity: Role of Global versus Local Hubs. This schematic illustrates the theoretical framework by Tao and Rapp (2021) for understanding how focal brain lesions affect network modularity. Lesions to global (connector) hubs that support inter‐modular communication are predicted to increase modularity by promoting segregation. In contrast, lesions to local (provincial) hubs that support intra‐modular integration are expected to reduce modularity. The figure highlights the differential consequences of disrupting distinct hub types on global network organization.

Tao and Rapp (2021) also emphasized that real lesions involve more than just node loss, showing that lesions lead to hub redistribution and functional reorganization (Figure 4C). This highlights the need to consider both immediate network disruption and long‐term reconfiguration in resilience models, providing a more comprehensive understanding of EOD progression and potential intervention strategies. Table 2A outlines how each graph metric is applied in the literature in EOD studies, distinguishing between general topological measures and those related to network resilience and Table 2B highlights clinical aspects that resilience metrics may help inform, such as subtype differentiation, compensatory processing, and potential links to cognitive reserve.

**FIGURE 4C:**
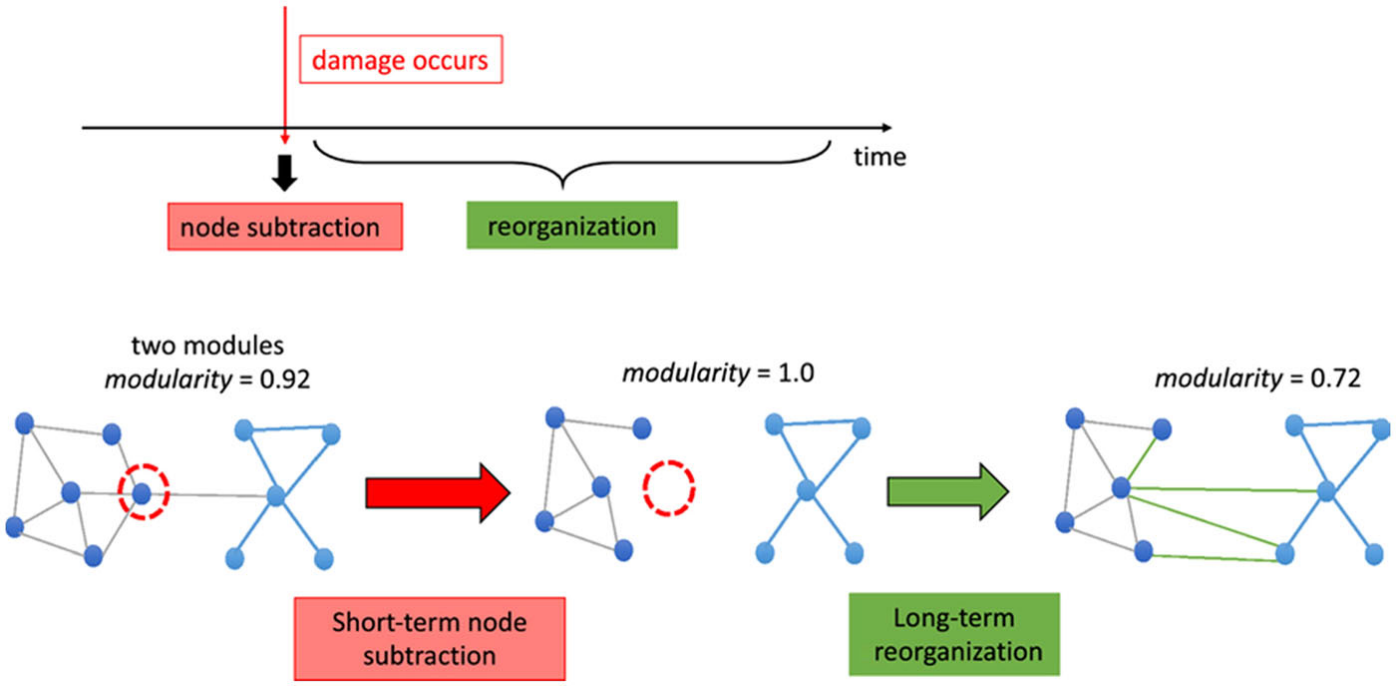
Two‐Stage Model of Lesion Effects: Node Subtraction versus Network Reorganization. This figure presents a conceptual model proposing that the functional consequences of focal brain lesions unfold in two distinct stages: (1) immediate structural impact through node and edge removal (“node subtraction”), and (2) delayed, adaptive network reorganization involving shifts in functional connectivity among spared regions. The model explains empirical discrepancies between simulated and real lesions, emphasizing the dynamic, plastic nature of the post‐lesion connectome. Figures 2 and 3A–C reprinted with permission from: Tao and Rapp (2021). Investigating the network consequences of focal brain lesions through comparisons of real and simulated lesions. *Scientific Reports, 11*(1), 2213. https://doi.org/10.1038/s41598‐021‐81107‐9.

**TABLE 2A:** From traditional graph metrics to resilience‐informed models in EOD.

Category	Metric	Used in reviewed EOD studies?	What it measures	Main limitation	What it misses/what resilience adds
Network integration	Global efficiency	✓Yes (widely)	Efficiency of information flow across the entire network	May be inflated by rerouting; does not capture structural fragility	Misses hidden vulnerability and early compensation/Simulates efficiency loss under node removal; reveals hidden vulnerability and collapse points
	Local efficiency	✓Yes	Regional fault tolerance		
	Small‐worldness	✓Yes	Optimal balance of integration and segregation	Can appear preserved despite network disintegration	Doesn't reflect dynamic compensation or collapse timing/Simulations can reveal when small‐world balance breaks down during progressive lesions
Modular organization	Modularity	✓Limited	Functional segregation into communities, Intra‐/inter‐modular connections	Rarely tracked over time or not tracked under attack	Can model modular degradation trajectories; may uncover subtype‐specific patterns
Hub architecture	Hub centrality (e.g., BC)	✓Yes	Key nodes for long‐range communication	Static snapshot; not much insights into reallocation	Misses Tao and Rapp's reorganization insights
	Rich club coefficient	✓Rare	Core network of high‐degree hubs	Underexplored in FTD; not linked to resilience	Can be tested with targeted attack to reveal backbone failure
Resilience models	LCC slope (Argiris et al. 2024)	✗ Not used	Global collapse rate under targeted attack	Not simulated in current literature	Dynamic index of network robustness, aspects of cognitive reserve
	Two‐stage lesion (Tao and Rapp 2021)	✗ Not used	Immediate damage + delayed reorganization	Absent in current literature	Explains compensation and paradoxes in EOD metrics
	Community resilience	✗ Not used	Integrity of modular systems under node loss	Not yet modeled in current literature	Identifies vulnerable networks and neuromodulation targets

**TABLE 2B:** Clinical aspects of EOD that network resilience metrics could potentially inform.

Clinical aspect	Potential contribution of resilience metrics
Cognitive reserve	Patterns of preserved network connectivity under simulated perturbation may serve as proxies for cognitive reserve, offering insight into compensatory brain capacity, and may predict individual differences in symptom severity and progression
Subtype differentiation, disease progression, and biomarker refinement	May help distinguish EOD subtypes, monitor changes in network integrity over time (e.g., via LCC decay), and assess the robustness of hubs or modules beyond static metrics.

### 5.2 Cortical Gradient Mapping: A Complementary Approach to Graph Network Analysis in EOD

In addition to graph‐theoretical approaches, cortical gradient mapping has emerged as a powerful technique for investigating macroscale functional network organization in neurodegenerative dementias, including EOD. Bouzigues et al. (2024) demonstrated that this method reveals a compression of the principal functional gradient, reflecting the unimodal‐to‐transmodal cortical hierarchy across FTD variants, such as bvFTD, svPPA, and nfvPPA. Their findings highlight widespread disruptions in bvFTD, particularly affecting the salience and DMNs, which extend beyond regions of structural atrophy and correlate with clinical symptoms like impaired social cognition.

Cortical gradient mapping offers a unique opportunity to capture continuous shifts in large‐scale network embedding, complementing structural and graph‐based analysis. By delineating how functional hierarchies dedifferentiate in EOD, this approach can uncover subtle network alterations that traditional metrics might overlook. For instance, the observed gradient compression in FTD underscores the breakdown of segregation between sensorimotor and transmodal networks, providing mechanistic insights into cognitive and behavioral deficits.

Integrating gradient mapping with graph theory could enhance our understanding of EOD pathophysiology. While graph metrics quantify topological disruptions (e.g., efficiency, modularity), gradient mapping contextualizes these changes within the brain's hierarchical organization. Future research should explore synergies between these methods, leveraging gradient‐derived features to refine resilience models or identify novel biomarkers. Such multimodal frameworks may ultimately improve early detection, subtype differentiation, and therapeutic targeting in EOD.

## 6 Conclusion

This review discusses graph‐theoretical findings across EEG, fMRI, DTI, and PET studies to characterize how network topology deteriorates in EOD, particularly FTD, bvFTD, and EOAD. Graph network analysis reveals the preservation of small‐world properties and compensatory mechanisms in early EOD, contrasting with a decline in efficiency and modular integration as the disease progresses. We identify a critical gap in current literature—the absence of resilience modeling: an approach that simulates network degradation under stress (e.g., LCC decay, targeted attacks) to reveal latent vulnerabilities. Future studies can move beyond static topology to uncover mechanistic signatures of network failure.

## Author Contributions

**Hema Nawani**: conceptualization, methodology, formal analysis, writing – review and editing, writing – original draft, data curation, visualization. **Sredha S. Sunil**: data curation. **Ranjith Jaganathan**: data curation, writing – review and editing. **Veeky Baths**: conceptualization, project administration, resources, writing – review and editing, writing – original draft, supervision, funding acquisition.

## Conflicts of Interest

The authors declare no conflicts of interest.

## Ethics Statement

The authors have nothing to report.

## Supporting information




**SupplementaryTable S1**: brb370903‐sup‐0001‐ TableS1.docx

## Data Availability

Data sharing is not applicable to this article, as no datasets were generated or analyzed during the current study. The DOIs of the studies included in the review are provided.
