# Supplementary material for: Brain Topology Disruption in Early‐Onset Dementia: Review of Current Findings and the Need for Network Resilience Focused Models
Source: Brain Behav. 2025 Nov 18;15(11):e70903. doi: 10.1002/brb3.70903 (PMC12627757; doi:10.1002/brb3.70903)
Supplement: Supplementary file 1 — SupplementaryTable S1: brb370903‐sup‐0001‐ TableS1.docx [file BRB3-15-e70903-s001.docx]

**Table 1 Fundamental Graph Metrics and Their Importance (Adapted from Tjims et al.^22^)**

| **Graph Characteristic** | **Description** | **Mathematical equation** | **Relevance** |
| --- | --- | --- | --- |
| **Node (Vertex)** | Fundamental unit representing objects or regions in the graph. The total number of nodes (vertices),$V$ defines the graph size. | $V=\sum_{i} v_{i}$  $v_{i}$ A single node; V: Total nodes in the graph. | Represents the building blocks of a network. |
| **Degree** | The number of edges (connections) a node has. | $\begin{aligned} Binary Values:k_{i}&=\sum_{j\in V} a_{ij}&\bar{k}&=\frac{1}{V}\sum_{i\in V} k_{i} \\ Weighted Values: k_{i}^{w}&=\sum_{j\in V} w_{ij}&\bar{k}^{w}&=\frac{1}{V}\sum_{i\in V} k_{i}^{w} \end{aligned}$  $a_{ij}:$ Binary adjacency (1 if nodes are connected, 0 otherwise); $w_{ij}:$Edge weight; $k_{i}$: Degree of node. ij: Node pairs i and j and their adjacency matrix | Identifies node connectivity and the overall connectivity pattern in the network |
| **Connectivity Density** | Ratio of actual edges to the maximum possible edges. | $S=\frac{\sum_{i\in V} k_{i}}{V(V-1)}$  $k_{i}$: Degree of node i; $k\left( V\left( V-1 \right) \right):$Maximum possible edges in a graph of size V. | Reflects network sparsity and overall resource efficiency. |
| **Path length** | Minimum number of edges connecting two nodes (i and j) | $L_{i}=\frac{\sum_{j\neq i\in V} L_{i,j}}{(V-1)} L=\frac{\sum_{i\in V} L_{i}}{V}$  $L_{i,j}$​: Shortest path between nodes i and j | Indicates the efficiency of communication across the network. |
| **Global Efficiency** | Inverse of the average shortest path length | $E_{\text{Global }}=\frac{1}{V(V-1)}\sum_{i\in V} \frac{1}{L_{i}}$  $L_{i}$: Path length for node i | Indicates overall integration of brain networks. |
| **Local Efficiency** | Efficiency of a node’s immediate neighborhood | $E_{\text{Local }}=\frac{1}{V}\sum_{i\in G} E\left( G_{i} \right)$  $G_{i}:$ Subgraph of the neighbors of node i | Reflects resilience to targeted attacks or failures in local brain circuits. |
| **Small-Worldness (σ)** | Describes networks with high clustering than random networks and path lengths equal to random network | $\sigma=\frac{C/\hat{C}}{L/\hat{L}}$  C and L are respectively the average clustering coefficient and average shortest path length;$\hat{C}$ and $\hat{L}$ are respectively the average clustering coefficient and average shortest path length of an equivalent random graph. | Indicates optimal information processing in brain networks. |
| **Betweenness Centrality** | Proportion of shortest paths (S) that pass through a specific node (i) and between node j and m | $BC_{i}=\sum_{i\neq j\neq m\in V} \frac{S_{j,m}\left( i \right)}{S_{j,m}}$  $BC=\frac{1}{V}\sum_{i\in V} BC_{i}$ | Identifies critical nodes ('hubs') facilitating long-distance communication in the network. |
| **Rich Club Coefficient** | Measures the connectivity among high-degree nodes. | $\Phi^{W}(k)=\frac{W_{>k}}{\sum_{l=1}^{E_{>}W_{l}^{\text{sorted }}}}$  The rich club coefficient of the weighted network, $\Phi^{W}(k)$, is computed through the ratio of W>k to the summation of the weights of the E>k strongest edges of the entire network (i.e. the top E>k edges in the sorted weight values Wsorted in descending order). | Indicates presence of a sub-network facilitating efficient global communication. |
| **Assortativity** | Measures the correlation between the degrees of connected nodes, representing the network’s preference for linking nodes of similar degree. | $r=\frac{\frac{1}{K}\sum_{i\in K} l_{i},m_{i}-\left( \frac{1}{K}\sum_{i\in K} \frac{1}{2}\left( l_{i}+m_{i} \right) \right)^{2}}{\frac{1}{K}\sum_{i\in K} \frac{1}{2}\left( l_{i}^{2}+m_{i}^{2} \right)-\left( \frac{1}{K}\sum_{i\in K} \frac{1}{2}\left( l_{i}+k_{i} \right) \right)^{2}}$  $K\text{ is the number of edges, }jl_{i}\text{ and }k_{mi}\text{ are the degrees of nodes connected by edge }i$ | Highlights the network's robustness and tendency to cluster similar nodes. |
| **Modularity** | Quantifies the division of the network into distinct communities or modules. | $Q_{m}^{w}=\sum_{s\in m} \frac{l_{s}}{L}-\left( \frac{d_{s}}{2L} \right)^{2}$  Here m is the number of modules, $l_{s}$ is the sum of weights in module s, L is the total sum of all weights in the network and $d_{s}$ is the sum of the strength of all vertices in module s | Indicates compartmentalizati- on and functional specialization. |
| \| **Network Resilience** Adapted from (Musawi et al., 2023, Argiris et al., 2024) \|  \|  \|  \|  \| \| --- \| --- \| --- \| --- \| --- \|  \|  \| Ability of communities to maintain intra- and inter-modular connectivity during node loss \| R_c = (1 / T) ∑ M(t) \| M(t): Modularity or number of connected communities at time t; T: steps of attack \| Tracks specialized community preservation \| \| --- \| --- \| --- \| --- \| --- \| \|  \|  \|  \|  \|  \| | Resistance to network breakdown under targeted attack. | $\begin{aligned} R_{n}=\frac{1}{\vert V\vert}\sum_{q=\frac{1}{\vert V\vert}}^{1} S(q) \end{aligned}$  $\begin{aligned} R_{e}=\frac{1}{\vert E\vert}\sum_{p=\frac{1}{\vert E\vert}}^{1} S(p) \end{aligned}$    Node Robustness (R_n_), Edge Robustness (R_e_) measures the connectivity of the network when subjected to the removal of nodes and links, respectively.  Here, \|V\| is the number of nodes, S(q) is the fraction of nodes in the largest connected subgraph after the removal of q\|V\| nodes (or p\|E\| edges), and q is the fraction of nodes to be targeted from the remaining nodes in \|V\| (or edges \|E\|). | Captures network robustness under progressive damage; higher R implies greater cognitive reserve. |
| **Community Resilience** Adapted from (Jao et al., 2019) | Preservation of modular integrity during node loss. | 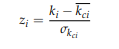 Intra-modular connectivity  where ki is the number of edges linking the ith node to other nodes in its module c; kci is the average of ki of all nodes in module c; and σkci is the standard deviation of the intra-modular degrees of all nodes in module c.  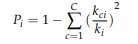Inter-modular connectivity  where kci is the number of edges connecting the ith node to other nodes in its module c, and ki is the number of degrees in node i in the network.  **Community resilience** reflects how well the **modular organization** of a network withstands progressive damage (e.g., node removal), calculated by tracking **how intra- and inter-community metrics change** during iterative attacks. | Reveals how intra- and inter-community structures resist disruption. |
